# Tuning the electrical and optical performance of PVA/PANI films *via* Fe_2_O_3_ nanorods for advanced energy storage and optoelectronic devices

**DOI:** 10.1039/d6ra01666a

**Published:** 2026-04-01

**Authors:** H. M. Ragab, N. S. Diab, Rosilah Ab Aziz, Shimaa Mohammed Aboelnaga, A. Al Ojeery, Tahani M. Alresheedi, M. O. Farea

**Affiliations:** a Basic Sciences Department, Foundation Year Program for Health and Medical Colleges, University of Ha'il Hail Saudi Arabia; b University of Jeddah, College of Science, Department of Physical Sciences Jeddah Saudi Arabia; c Department of Chemistry, College of Science, Qassim University Buraydah Saudi Arabia; d Physics Department, Faculty of Science, Ibb University Ibb Yemen

## Abstract

PVA/PANI-Fe_2_O_3_ nanocomposite films were successfully fabricated *via* a solution casting method with Fe_2_O_3_ nanorod loadings ranging from 1.0 to 4.5 wt% to tailor their structural, optical, and electrical properties for optoelectronic applications. XRD analysis revealed a progressive reduction in crystallite size (from ∼4.2 to ∼3.0 nm) and enhanced amorphous character, attributed to the disruption of the native hydrogen-bonding network in the polymer matrix by Fe_2_O_3_ nanorods. FTIR results confirmed strong interfacial interactions through hydrogen bonding between Fe_2_O_3_ and PVA/PANI chains. Optical measurements demonstrated a red shift in absorption edges and a significant narrowing of both direct and indirect band gaps, accompanied by an increase in Urbach energy, indicating the formation of localized states and increased structural disorder. These changes are associated with the modified electronic structure induced by Fe_2_O_3_ incorporation. Furthermore, dielectric analysis revealed multiple conduction regimes, with electrical conductivity improving by nearly two orders of magnitude due to the formation of interconnected charge transport pathways facilitated by the nanorods. Overall, the incorporation of Fe_2_O_3_ nanorods effectively tunes the physicochemical properties of PVA/PANI films, making them promising candidates for flexible optoelectronic and energy-related applications.

## Abstract 2

PVA/PANI-Fe_2_O_3_ nanocomposite films were successfully fabricated *via* a solution casting method with Fe_2_O_3_ nanorod loadings ranging from 1.0 to 4.5 wt% to tailor their structural, optical, and electrical properties for optoelectronic applications.

## Graphical abstract

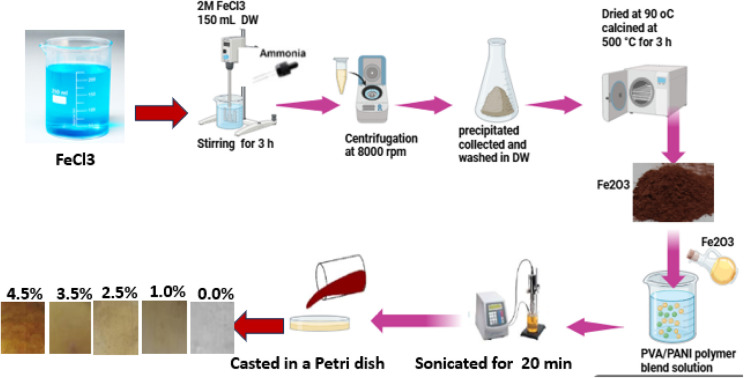

## 1 ntroduction

Polymer nanocomposites have become essential in modern technological development and play a significant role in various aspects of daily life.^1–3^ Recent research primarily focuses on designing innovative polymer nanocomposites with improved physical and functional properties.^4,5^ These polymers are highly attractive for both scientific and industrial applications due to their affordability, biodegradability, ease of fabrication, and favorable optoelectronic characteristics.^6–9^ Poly(vinyl alcohol) is a bio-compatible and thermally stable material,^10^ that has been widely employed as a base material for diverse nanofiller additives due to its advantageous properties, including water solubility, non-corrosive behavior, excellent film-forming ability, and high optical transparency. In our previous studies, a range of nanoparticles, including CoFe_2_O_4_.^11^ Bi_2_O_3_/MWCNT hybrid nanofillers,^12^ Al_2_O_3_/V_2_O_5_ nanoparticles,^13^ and niobium oxide and praseodymium oxide nanoparticles^14^ have been incorporated into PVA matrices to tailor and enhance their optical properties for potential industrial applications. In addition, polyaniline (PANI), a conducting polymer with semiconducting or metallic behavior,^15^ has attracted attention due to its good electrical conductivity, electrochemical performance, stability, accessibility, and low-cost synthesis. Laourari *et al.* synthesized NiCu-PANI/PVA quaternary nanocomposite films and demonstrated their strong antibacterial performance and Cu-enhanced antifungal activity against selected pathogens.^16^ Li *et al.*^17^ synthesized a flexible APH-PANi hydrogel sample with vertically aligned channels that serves as an integrated, self-healable supercapacitor exhibiting high capacitance, excellent mechanical flexibility, and stable electrochemical performance. Abdelhamied *et al.*^18^ prepared PVA/PANI/Ag nanocomposite films and showed that oxygen-ion irradiation significantly modifies their structural, linear, and nonlinear optical properties, with the highest fluence producing films most suitable for optoelectronic applications. Arenas *et al.*^19^ synthesized PVA-PANI *in situ* nanocomposite films using surfactant and acid dopants, demonstrating low percolation thresholds and enhanced electrical conductivity suitable for antistatic electronic applications. Alsulami and Rajeh^20^ fabricated PANI/PMMA-TiO_2_ nanocomposite films and demonstrated that increasing TiO_2_ doping enhances thermal stability and significantly modifies the optical properties, making the films promising for optoelectronic applications.

Magnetic nanoparticles have attracted considerable attention as nanofillers due to their distinctive properties, including enhanced performance relative to bulk materials and the ability to be precisely controlled by external magnetic field.^21^ Among magnetic iron nanoparticles, Fe_2_O_3_ (hematite) is particularly appealing because it combines exceptional corrosion resistance, cost-effective synthesis, bio-compatibility, and eco-friendly due to its non-toxic nature.^22^ Furthermore, hematite nanoparticles exhibit a high surface-to-volume ratio, which results in elevated surface energy and enhanced reactivity.^23^ By incorporating these nanoparticles into polymer matrices, their unique characteristics can be exploited to improve the versatility and physicochemical properties of the resulting nanocomposite materials.^24–26^ Badawi *et al.*^27^ prepared PVA/graphene nanocomposite films loaded with Fe_2_O_3_ nanoparticles and demonstrated that varying the iron oxide content significantly tailors their structural and optical parameters, making them suitable for diverse optical and storage applications. Ragab *et al.*^28^ fabricated PEO@NaAlg sample reinforced with Fe_2_O_3_ nanorods and showed that increasing nanorod content significantly modifies their optical, magnetic, and electrical properties, enhancing their suitability for magneto-optical and energy-storage applications. El Sayed and Morsi^29^ fabricated α-Fe_2_O_3_-reinforced (PVA + PEG) nanocomposite films and demonstrated that increasing hematite content significantly tunes their optical and dielectric properties through enhanced refractive index, reduced band gap, and improved AC conductivity.

To address this gap, the present study focuses on the incorporation of α-Fe_2_O_3_ nanorods into PVA/PANI hybrid nanocomposites and investigates how their unique rod-like morphology influences the structural, optical, and electrical properties. Despite extensive studies on PVA/PANI composites with metal oxides, limited attention has been given to the role of nanorod morphology in simultaneously tuning the optical band gap, Urbach energy, and AC/DC conductivity. In this work, α-Fe_2_O_3_ nanorods with high aspect ratio were incorporated over a controlled loading range (1–4.5 wt%), allowing a systematic correlation between nanorod dispersion, structure, and multifunctional properties. The main findings demonstrate that Fe_2_O_3_ nanorods enhance the amorphous nature of the polymer matrix, reduce the optical band gap, increase Urbach energy, and significantly improve electrical conductivity through efficient charge transport pathways. These results highlight the novelty of this work and underscore the strong potential of PVA/PANI-Fe_2_O_3_ nanocomposites as tunable multifunctional materials for advanced optoelectronic and energy-related applications.

## 2 Methodology

### 2.1 Chemicals

Polyvinyl alcohol (PVA) with an average molecular weight of about 75 000 g mol^−1^ was purchased from Merck (Germany). Polyaniline (PANI), having a molecular weight exceeding 19 000 g mol^−1^, was sourced from Sigma-Aldrich. Ferric chloride (FeCl_3_, ≥98% purity, Sigma-Aldrich), deionized water, and aqueous ammonia solution (NH_4_OH, 25–28%, Sigma-Aldrich) were used in the preparation of the Fe_2_O_3_ nanorods.

### 2.2 Synthesis of Fe_2_O_3_ nanorods

Fe_2_O_3_ nanorods were synthesized using a co-precipitation approach. A 2 M FeCl_3_ solution was prepared in 150 mL of deionized water and heated to 90 °C. Ammonia was gradually introduced under continuous stirring for 3 h to induce precipitation. The resulting product was centrifuged at 8000 rpm, and the obtained precipitate was repeatedly washed with deionized water. The Fe_2_O_3_ nanorods were then dried at 90 °C for 48 h and subsequently calcined at 500 °C for 3 h.

### 2.3 Preparation of PVA/PANI-Fe_2_O_3_ nanocomposites

PVA/PANI blend films with an 80 : 20 ratio was fabricated using the solution casting technique. First, 1.6 g of PVA was dissolved in 100 mL of deionized water at 80 °C under continuous stirring until a clear homogeneous solution was formed. Separately, 0.4 g of PANI was dispersed in 40 mL of deionized water at room temperature. The two solutions were then combined, as the 80 : 20 PVA/PANI proportion was found to yield highly uniform and flexible films. The blended mixture was stirred for an additional hour to ensure complete homogenization. Fe_2_O_3_ nanorods were incorporated into the polymer blend at various loadings (1.0, 2.5, 3.5, and 4.5 wt%). Each amount of nanofiller was incorporated gradually to the solution under constant stirring for 1 hour, followed by ultrasonic treatment for 20 min at room temperature to minimize particle agglomeration. The required nanorod content M (1.0–4.5 wt%) was computed by eqn (1):1
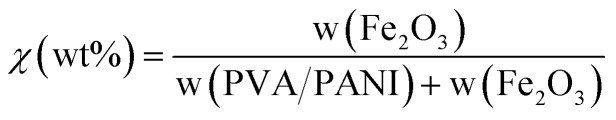


**Scheme 1:**
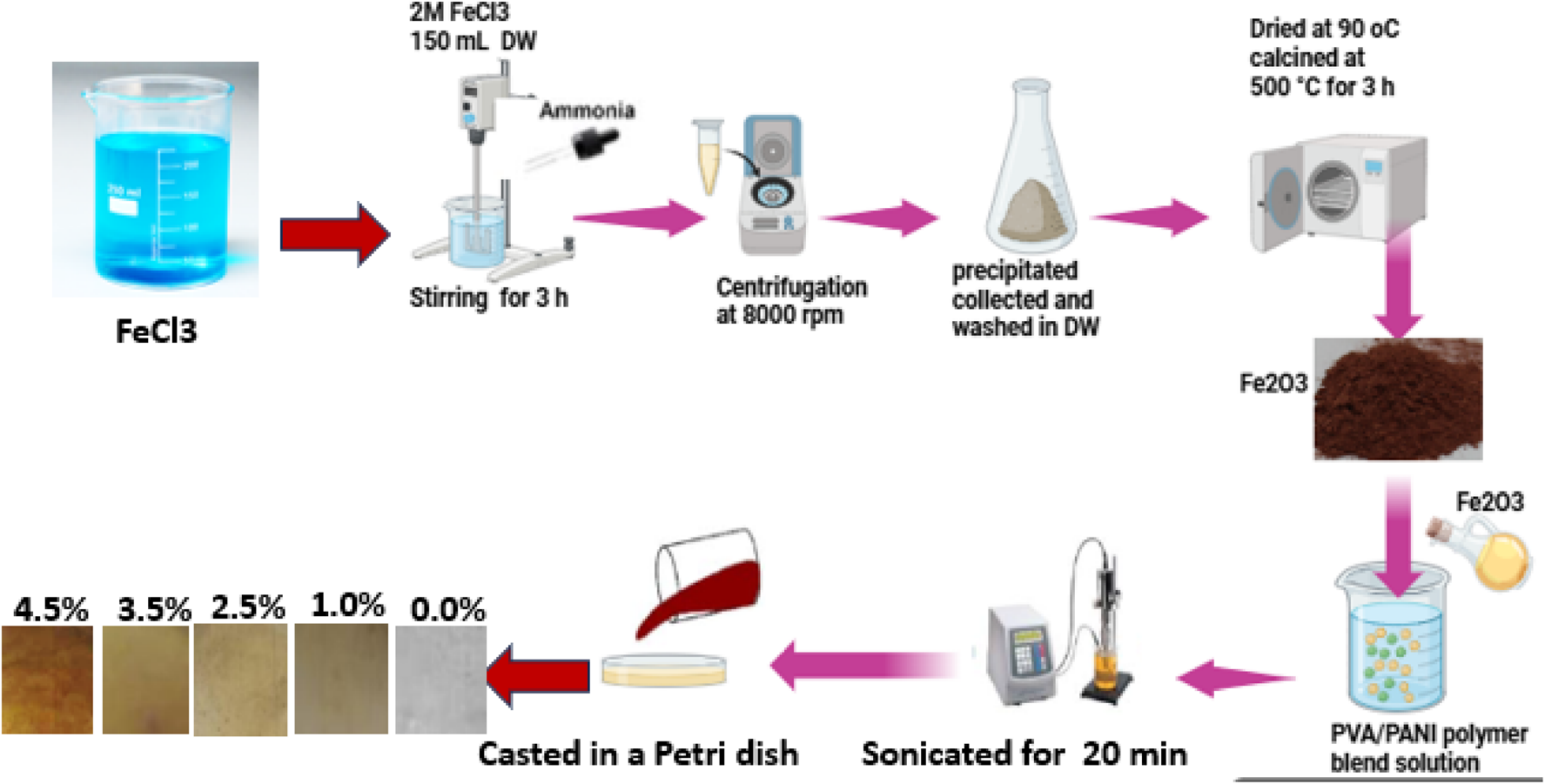
A schematic illustration of the synthesis procedure of the PVA/PANI-Fe_2_O_3_ nanocomposites films.

Finally, the prepared mixtures were cast into Petri dishes and allowed to dry at 55 °C for four days. Uniform films with an approximate thickness of 0.08 mm were obtained by gently peeling them from the dishes. A schematic illustration of the synthesis procedure is presented in Scheme 1, which outlines the formation process of the PVA/PANI-Fe_2_O_3_ nanocomposites films.

### 2.4 Device characterizations

The structural, optical, and electrical properties of the PVA/PANI-Fe_2_O_3_ nanocomposites were characterized using a range of analytical techniques. X-ray diffraction (XRD) patterns were recorded using a PANalytical X'Pert Pro diffractometer with Cu Kα radiation (*λ* = 0.15406 nm), operating at 40 kV and 30 mA, over a 2*θ* range of 3–70°. FTIR spectra were recorded using a Bruker Vertex 80 spectrometer in the range of 400–4000 cm^−1^. Optical absorption spectra were obtained using a Shimadzu UV-3600 spectrophotometer operating between 200 and 1000 nm under ambient conditions. Dielectric properties were measured by broadband dielectric spectroscopy using a Novocontrol Alpha-A Analyzer within the frequency range of 0.1–10 MHz.

## 3 Results and discussion

### 3.1 XRD analysis

**Fig. 1:**
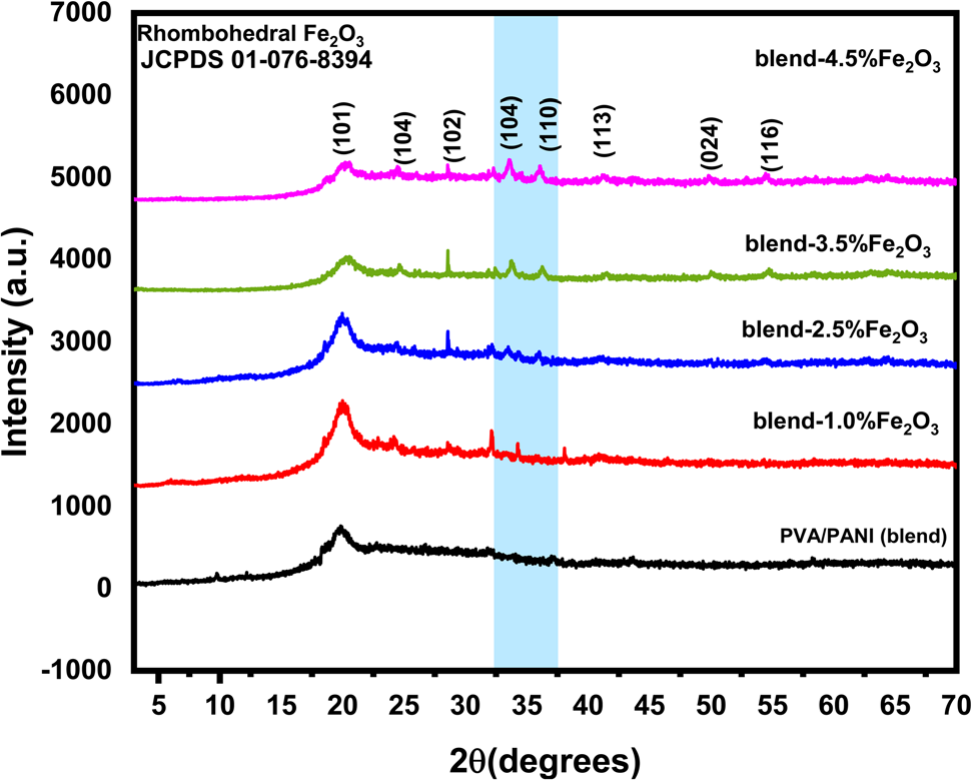
XRD patterns for pure PVA/PANI polymer blend and PVA/PANI-Fe_2_O_3_ nanocomposites samples.

**Fig. 2:**
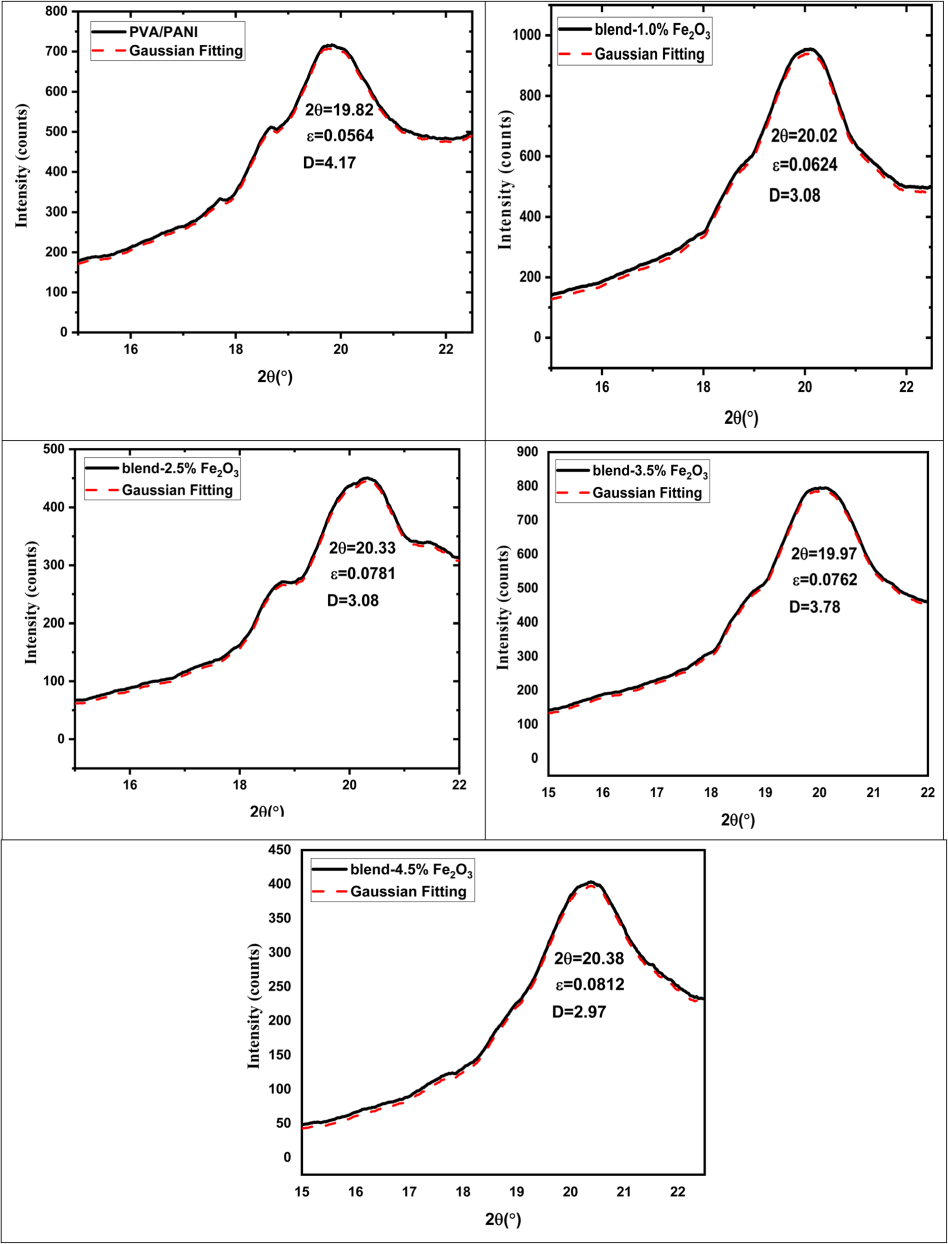
Gaussian fitting of (101) peak for pure PVA/PANI polymer blend and PVA/PANI-Fe_2_O_3_ nanocomposites samples.

Fig. 1 presents the XRD patterns of the pure PVA/PANI blend alongside those of the nanocomposite films containing various loadings of Fe_2_O_3_ nanorods (NRs). The broad diffraction halo centered at 19.73°, corresponding to the (101) plane, is typical of semi-crystalline polymer blend. Upon incorporating Fe_2_O_3_ NRs into the PVA/PANI polymer blend, this halo becomes progressively wider and less intense, indicating a reduction in structural order as the nanorod content increases. Additionally, diffraction peaks appearing at 33.76° and 36.10°, assigned to rhombohedral Fe_2_O_3_ (JCPDS 01-076-8394), are detected and become increasingly distinct at higher nanorod concentrations.^30^ This behavior signifies that introducing Fe_2_O_3_ NRs enhances the amorphous character of the PVA/PANI blend.^31^ The more noticeable Fe_2_O_3_ reflections at elevated filler content, along with their absence at lower levels, suggest an increase in particle size due to agglomeration as the Fe_2_O_3_ loading rises. The interactions between Fe_2_O_3_ nanorods and PVA/PANI chains are further supported by the formation of hydrogen bonds between the nanorods and the hydroxyl groups of PVA/PANI. A similar trend was observed by Abdelhamied *et al.*,^18^ who observed a decrease in semicrystallinity when Ag nanoparticles were incorporated into PVA/PANI polymer blend. To quantify structural parameters, the main (101) peak of the PVA/PANI-based samples was fitted using a Gaussian function (Fig. 2). From this analysis, the values of (*D* nm), (*ε*), and (*δ*) were determined using the standard relations provided in ref. 32:2
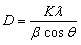
3
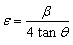
4
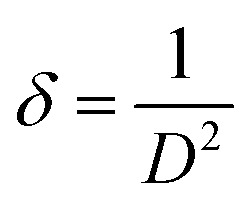


The calculated structural parameters are summarized in Table 1 and illustrated in Fig. 2. It is evident that the crystallite size (*D*) decreases progressively as the Fe_2_O_3_ nanorod content in the PVA/PANI matrix increases. This reduction is attributed to the disruption of the original hydrogen-bonding network within the PVA/PANI blend when Fe_2_O_3_ nanorods are introduced, as new hydrogen bonds form between the nanorods and the polymer chains instead.^33^ Conversely, both the internal strain (*ε*) and dislocation density (*δ*) exhibit an upward trend with increasing Fe_2_O_3_ nanorod concentrations. The elevated values of *ε* and *δ* further support the conclusion that the crystallinity of the PVA/PANI blend diminishes upon incorporation of Fe_2_O_3_ nanorods.^34^

**Table 1:** Geometrical parameters of PVA/PANI-Fe_2_O_3_ nanocomposites samples

Composites	*β* (radians)	2*θ*	*D* (nm)	*ε*	*δ* (nm^−2^)
PVA/PANI (blend)	2.65	19.82	4.20	0.0564	0.124
Blend-1.0% Fe_2_O_3_	2.87	20.02	3.3	0.0624	0.146
Blend-2.5% Fe_2_O_3_	3.14	20.33	3.2	0.0781	0.152
Blend-3.5% Fe_2_O_3_	3.49	19.97	3.1	0.0762	0.178
Blend-4.5% Fe_2_O_3_	3.38	20.38	3.0	0.0812	0.169

### 3.2 FTIR study

**Fig. 3:**
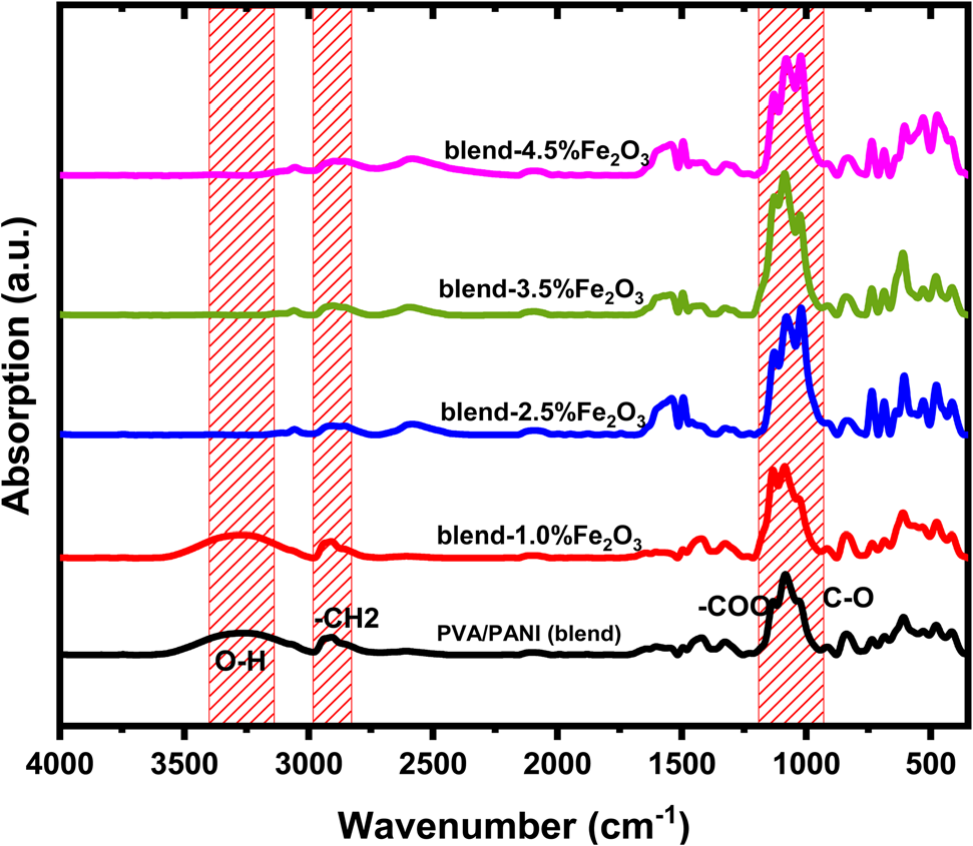
FT-IR spectra of the PVA/PANI blend and the nanocomposite films containing various loadings of Fe_2_O_3_ nanorods (NRs).

**Scheme 2:**
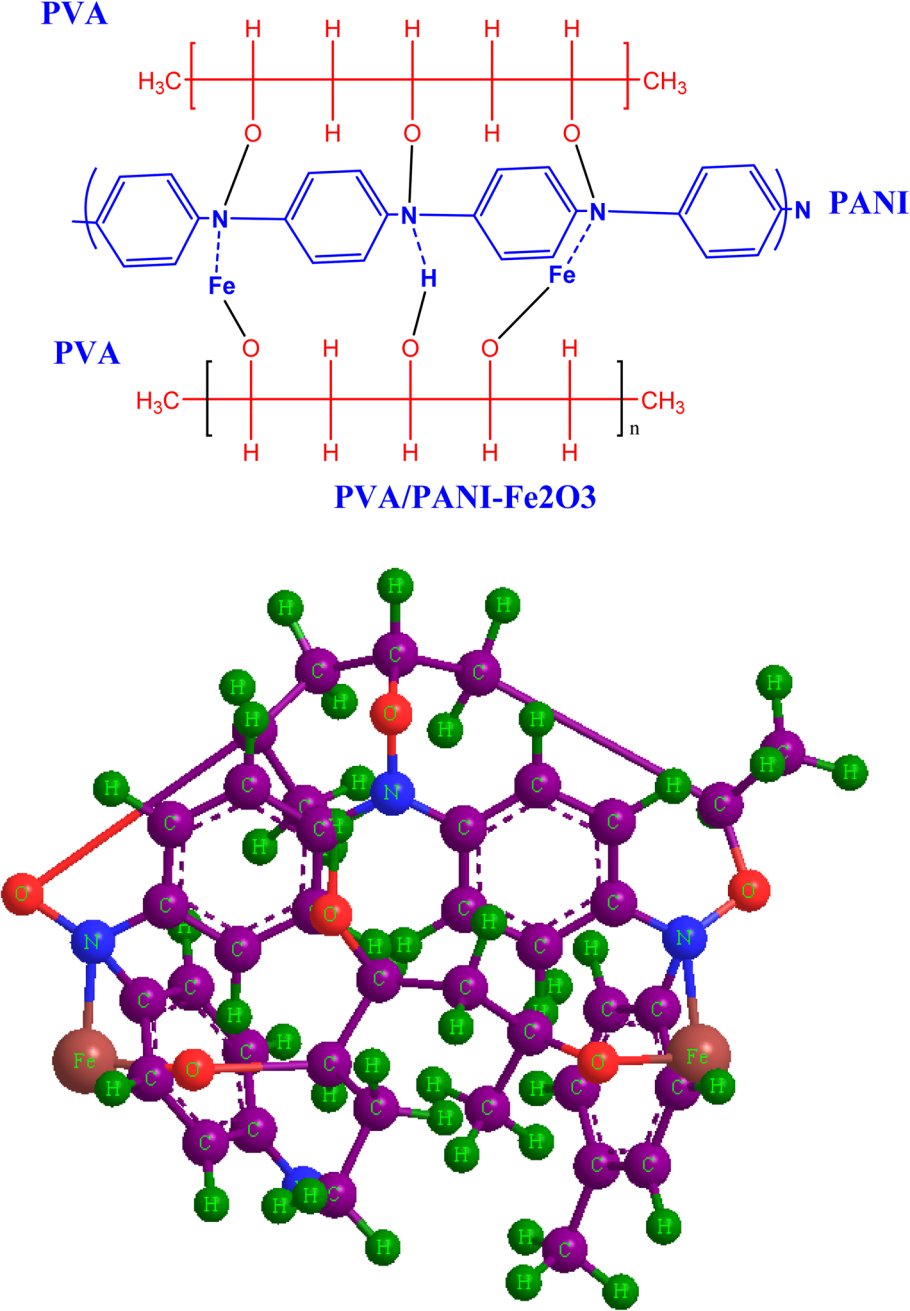
Schematic representation of the proposed interaction mechanism between PVA/PANI and Fe_2_O_3_ nanorods, illustrated in both 2D and 3D formats.

FTIR spectroscopy was employed to examine how the functional groups within the PVA/PANI polymer matrix interact with the incorporated Fe_2_O_3_ nanorods. Fig. 3 shows the FT-IR spectra of pristine PVA/PANI and composites containing different concentrations of Fe_2_O_3_ nanorods. In the pristine PVA/PANI sample, a broad and intense absorption band appears at 3286 cm^−1^, which is ascribed to OH stretching vibration, confirming the presence of hydroxyl group.^35^ As the loading of Fe_2_O_3_ nanorods increases, this band progressively diminishes and eventually disappears, indicating the creation of intermolecular hydrogen bond between the OH group of poly(vinyl alcohol) and the Fe_2_O_3_ surface.^36^ This modification implies that the CH_2_ units in the side chain of PVA/PANI may coordinate with Fe^2+^ ions. The absorption at 2933 cm^−1^ corresponds to the asymmetric stretching of CH_3_ groups.^37^ Additionally, the pure PVA/PANI exhibits a peak at 1609 cm^−1^, associated with the stretching of the –COO functional group. The band at 1481 cm^−1^ is linked to CH_2_ scissoring vibrations, while the signal at 1318 cm^−1^ arises from ether group stretching. The characteristic C–O stretching vibration appears near 1134 cm^−1^,^38^ and the peak detected at 1084 is due to asymmetric C–C stretching modes.^37^ Another notable peak at 858 corresponds to CH_2_ stretching vibrations.^36^ The incorporation of Fe_2_O_3_ nanorods into the polymer blend caused noticeable shifts in key absorption peaks, particularly those at 3286, 1609, and 1134 cm^−1^, toward lower wavenumbers, confirming strong interactions between the nanofiller and the host matrix. Minor spectral changes also emerged with increasing Fe_2_O_3_ content, consistent with modifications in the polymer environment upon doping. The overall reduction in characteristic vibrational frequencies provides clear evidence of bonding between PVA/PANI and Fe_2_O_3_ nanorods, as illustrated in Scheme 2. These interactions contribute to a decrease in the crystallin degree within the amorphous region, in agreement with the XRD results.

### 3.3 TEM image

**Fig. 4:**
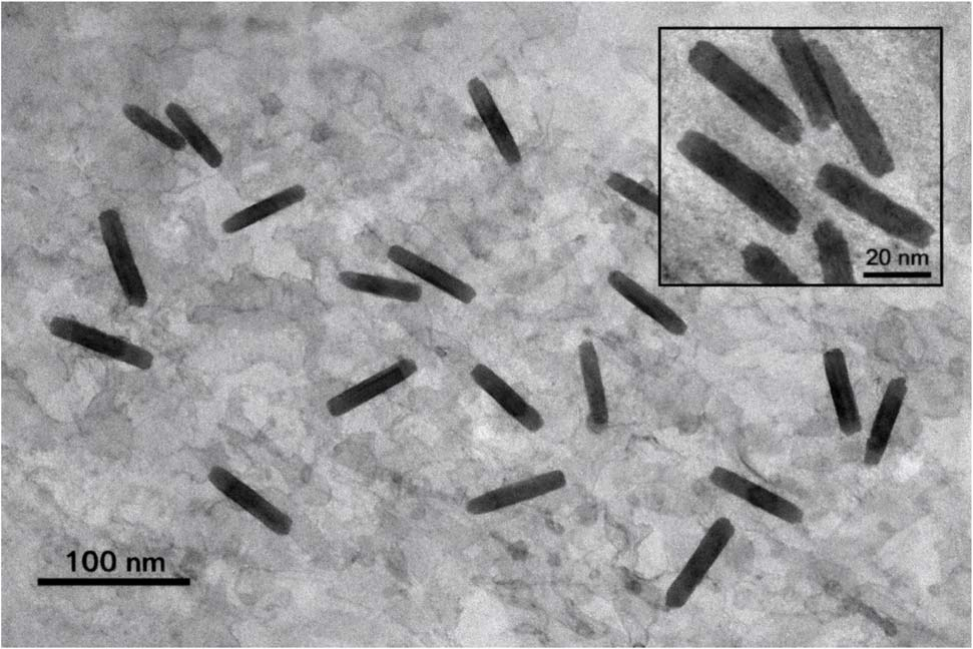
TEM image of PVA/PANI-Fe_2_O_3_ (4.5 wt%) showing well-dispersed nanorods with slight aggregation; inset shows higher magnification.

The TEM image corresponding to the highest Fe_2_O_3_ nanorod loading (4.5 wt%) reveals a dense and well-dispersed distribution of nanorods within the PVA/PANI polymer matrix (Fig. 4). The nanorods exhibit a distinct elongated morphology with a high aspect ratio, confirming the successful synthesis of rod-like Fe_2_O_3_ structures *via* the co-precipitation method.^39^ At this concentration, the nanorods are closely packed, forming a semi-interconnected network throughout the polymer matrix, which is expected to facilitate charge transport pathways. Despite the increased filler content, the nanorods remain relatively uniformly distributed due to the combined effect of prolonged stirring and ultrasonication, although slight agglomeration can be observed in some localized regions. This homogeneous dispersion with limited aggregation indicates strong interfacial interactions between Fe_2_O_3_ nanorods and the PVA/PANI chains, which plays a crucial role in enhancing the electrical conductivity and optical performance of the nanocomposite films.

### 3.4 UV-visible spectroscopy

**Fig. 5:**
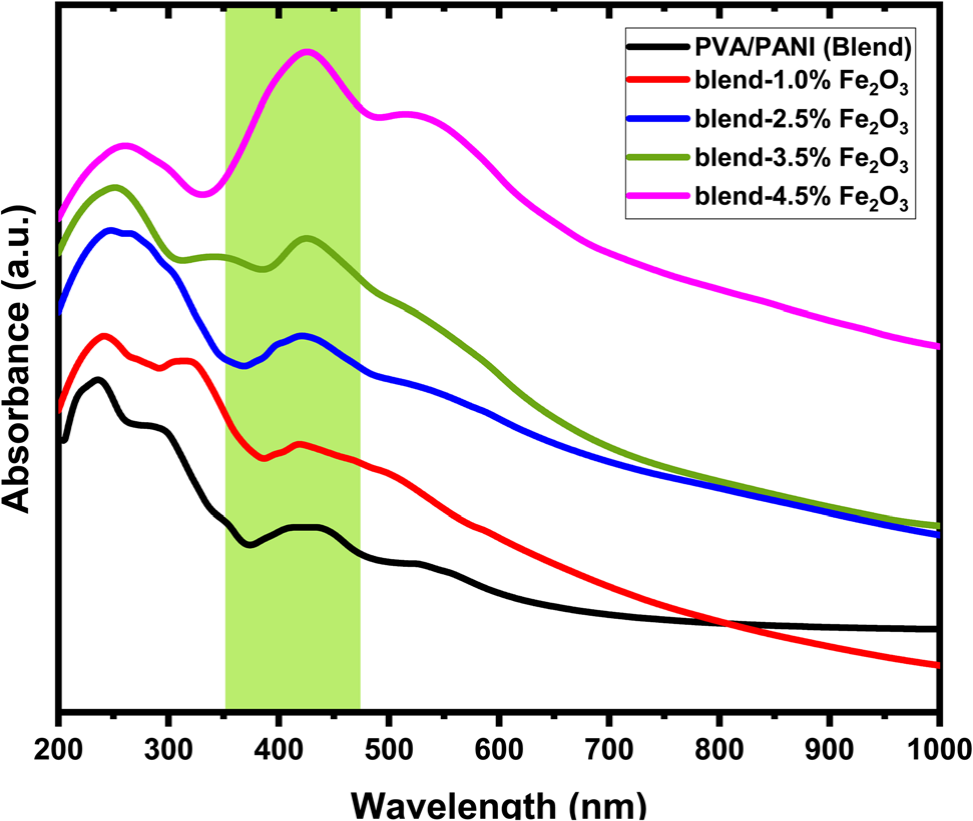
The plots of absorption *versus* wavelength for PVA/PANI matrix and the nanocomposite films containing various loadings of Fe_2_O_3_ nanorods.

The optical behavior of the prepared samples was examined using UV-visible spectroscopy. The corresponding spectra for the pristine PVA/PANI blend and the Fe_2_O_3_-incorporated PVA/PANI nanocomposites are presented in Fig. 5. In the UV region of the PVA/PANI spectrum, medium-intensity absorption bands appear at approximately 233, 292, and 398 nm, which are commonly associated with the carbonyl functionalities of PVA.^40^ Also, these bands originate from the π → π* electronic transitions of the benzenoid and quinoid segments of PANI. Upon introducing Fe_2_O_3_ nanorods into the polymer matrix, the ternary nanocomposites exhibit a noticeable red shift in the characteristic absorption peaks of the PVA/PANI blend. Furthermore, samples loaded with 2.5%, 3.5%, and 4.5% Fe_2_O_3_ show a marked increase in absorbance within the UV region.^41^ This shift is indicative of strong interactions between the Fe_2_O_3_ nanorods and the PVA/PANI host polymer. An additional absorption feature observed around 531 nm is attributed to the presence of iron oxide nanorods.^42^

### 3.5 Optical parameters

#### 3.5.1 Energy gap

The energy gap (*E*_g_) of the PVA/PANI-Fe_2_O_3_ nanocomposites was calculate by the Davis–Mott equation:^43^

5 (*αhυ*) = *Q*(*hυ* − *E*_g_)^*r*^

**Fig. 6:**
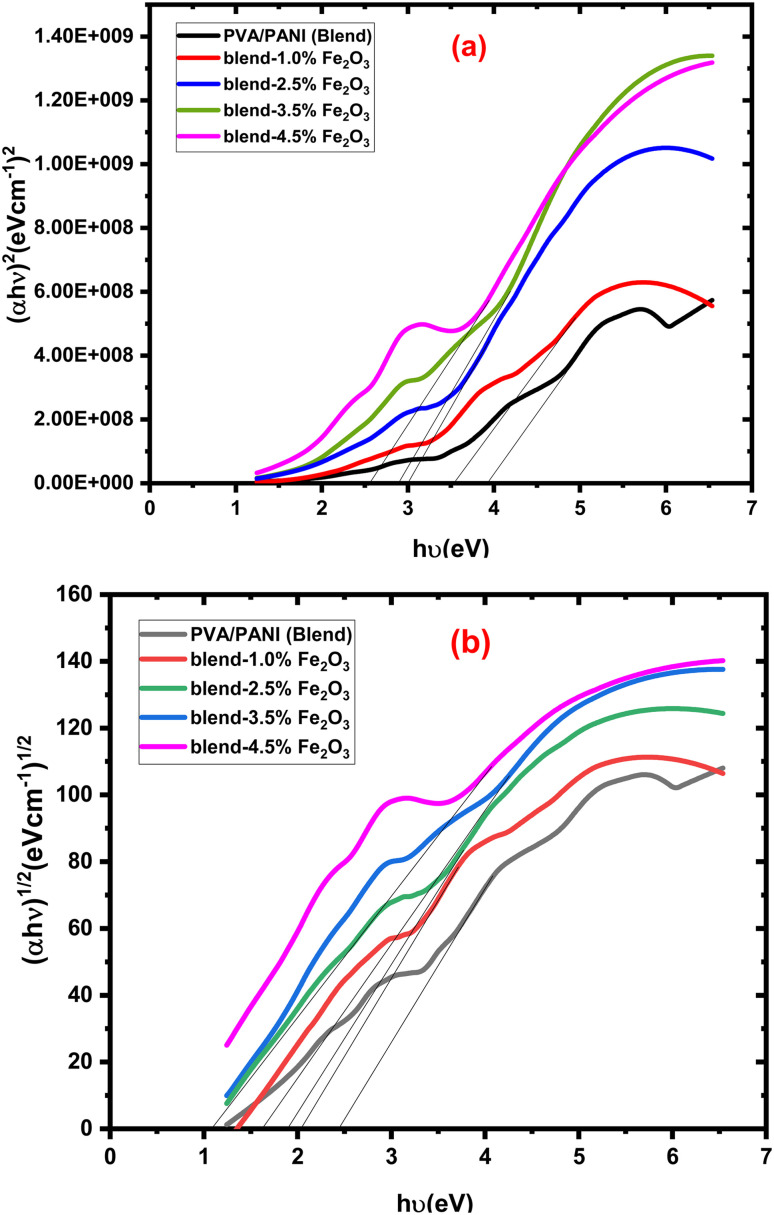
The relationship between (a) (*αhυ*)^2^ and (b) (*αhυ*)^1/2^ and *hυ* for PVA/PANI-Fe_2_O_3_ nanocomposites samples.

Here *α*(cm^−1^) is the absorption coefficient, *hν* (eV) is the photon energy, and *r* takes the values 2 and 1/2 for direct (*E*_gd_) (eV) and indirect (*E*_gi_) (eV) allowed transitions, respectively. Here, *E*_g_ denotes the optical band gap, while *C* is a constant related to the absorption edge. The extracted *E*_gd_ and *E*_gi_ values, calculated from the plots in Fig. 6(a and b), are summarized in Table 2. A progressive reduction in both the direct and indirect band gap energies is observed with increasing Fe_2_O_3_ loading in the PVA/PANI matrix. This trend, evident in Fig. 6 and Tables 2, is ascribed to the creation of charge transfer complexes between the Fe_2_O_3_ nanorods and the functional group of the PVA/PANI blend, which modifies the electronic structure and narrows the band gap. Additionally, the incorporation of nanofillers introduces structural defects that generate localized states within the band gap, further contributing to the observed decrease in *E*_g_.^44^ Furthermore, the observed band gap narrowing can be attributed to the combined effect of interfacial interactions, defect-induced localized states, and charge transfer mechanisms. The strong interfacial interaction between α-Fe_2_O_3_ nanorods and the PVA/PANI chains leads to electronic coupling, which modifies the density of states near the band edges. In addition, the incorporation of Fe_2_O_3_ introduces structural disorder and defect levels within the forbidden band gap, acting as intermediate energy states that facilitate electronic transitions at lower photon energies.

**Table 2:** The optical and electrical parameters for PVA/PANI-Fe_2_O_3_ nanocomposite films

Composites	*E*_gd_ (eV)	*E*_gi_ (eV)	*E*_u_ (eV)	*σ*_dc (S.cm_^−1^_)_	*σ*_ac (S.cm_^−1^_)_
PVA/PANI (blend)	3.91	2.45	0.1736 ± 0.0014	3.58 × 10^−13^	5.39 × 10^−8^
Blend-1.0% Fe_2_O_3_	3.53	2.04	0.1922 ± 0.0037	4.09 × 10^−12^	1.14 × 10^−7^
Blend-2.5% Fe_2_O_3_	3.01	1.91	0.2185 ± 0.0027	1.48 × 10^−11^	5.89 × 10^−7^
Blend-3.5% Fe_2_O_3_	2.90	1.63	0.2318 ± 0.0052	6.41 × 10^−11^	9.92 × 10^−7^
Blend-4.5% Fe_2_O_3_	2.59	1.09	0.2537 ± 0.0034	2.64 × 10^−10^	3.32 × 10^−6^

#### 3.5.2 Urbach energy

**Fig. 7:**
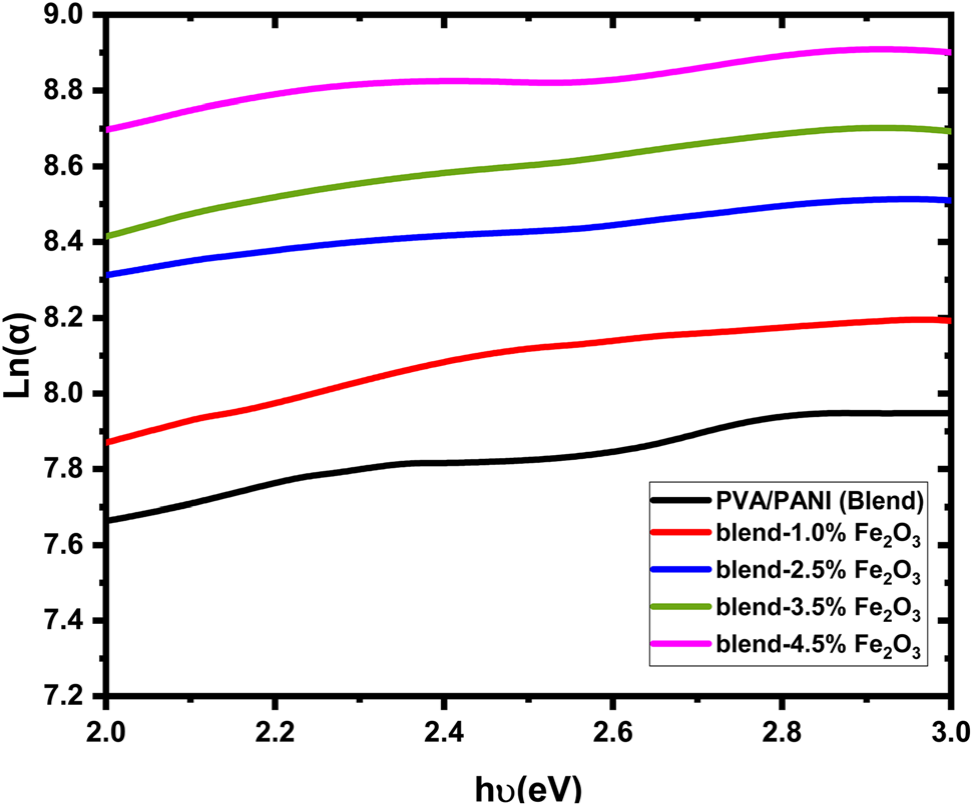
The plots of ln(α) *vs.* photon energy for pure PVA/PANI polymer blend and PVA/PANI-Fe_2_O_3_ nanocomposites samples.

The incorporation of Fe_2_O_3_ nanorods increases the structural disorder within the polymeric matrix. This enhancement in disorder can be evaluated by estimating the Urbach energy (*E*_U_), which is derived from the empirical relationship between the absorption coefficient and photon energy:^45^6
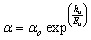
where *α*_o_ (cm^−1^) is a material-dependent constant. Fig. 7 presents the plot of ln(α) Vs. *hυ* in the region near the absorption edge, yielding a linear dependence consistent with eqn (6). The Urbach energy values, determined from the inverse slope of these linear fits, are listed in Table 2. An increase in *E*_U_ is observed with higher Fe_2_O_3_ content in the PVA/PANI matrix, indicating that the introduction of nanofiller enhances structural disorder and results in the creation of additional localized states inside the band gap. This broadening of the tail states increases the probability of electronic transitions such as tail-to-tail and band-to-tail, further reflecting the higher degree of disorder in the prepared nanocomposite films.

### 3.6 Electrical properties

#### 3.6.1 AC conductivity

**Fig. 8:**
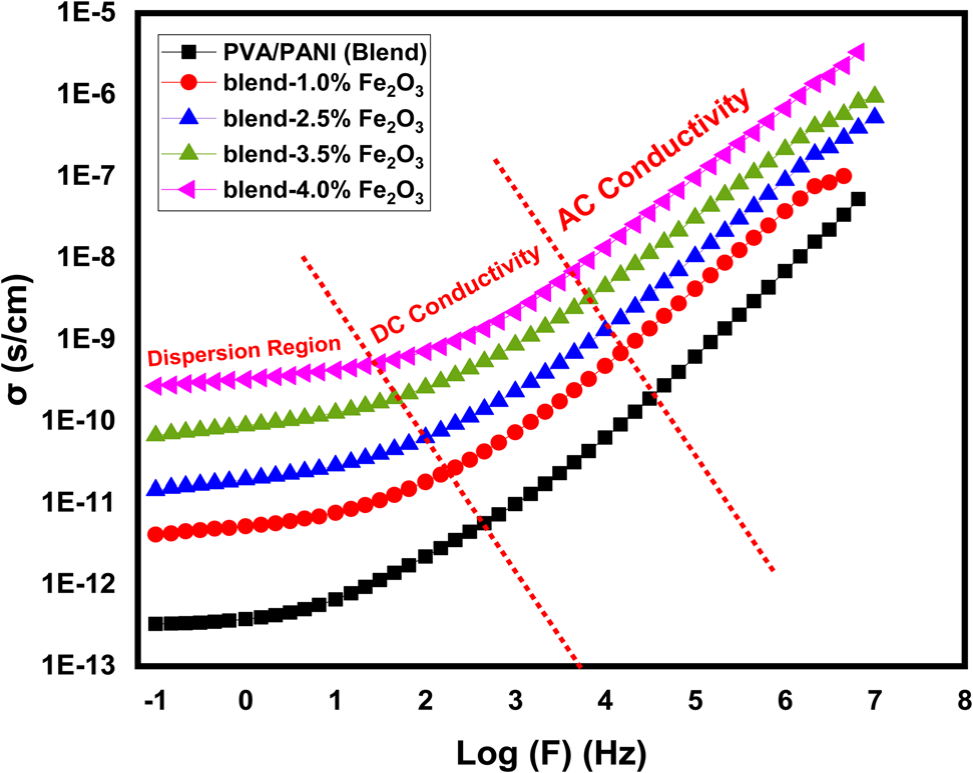
The plots of *σ*_ac_*versus* log (*F*) for pure PVA/PANI polymer blend and PVA/PANI-Fe_2_O_3_ nanocomposites samples.

The electrical conductivity of materials, especially their alternating current conductivity (*σ*_ac_), plays a vital role in explaining charge transport behavior in polymer nanocomposites.^46^ The electrical conductivity of the PVA/PANI system containing different loadings of Fe_2_O_3_ nanorods was evaluated at ambient temperature over a wide frequency window, as displayed in Fig. 8. The conductivity values were calculated using eqn (7):^47^7
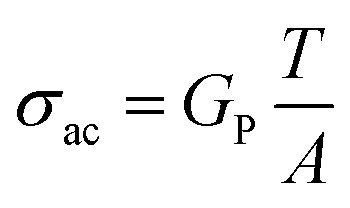
Here *G*_p_ (S (Siemens)) is the parallel conductance of the film, *A* (cm^2^) its surface area, and *T*(cm) is the film thickness. The conductivity curves reveal three characteristic frequency-dependent regions. Low-frequency region (dispersion): *σ*_ac_ shows a reduction due to electrode polarization. In this regime, charge carriers gather at the electrode/electrolyte interface, forming a barrier and restricting charge movement. The conductivity remains almost constant here since charge transport is mainly controlled by freely moving ions within the polymer matrix. Intermediate frequency region (*σ*_dc_ plateau): conductivity approaches the DC limit (*σ*_dc_), attributed to ions migrating through the bulk of the material.^48^ This plateau reflects the intrinsic ion-transport capability of the polymeric matrix under an applied electric field. High-frequency region (rise in *σ*_ac_): conductivity increases sharply, signifying improved carrier mobility. This behavior is associated with the release or activation of trapped ions, which can readily follow the rapidly alternating field at higher frequencies.^49^ Introducing Fe_2_O_3_ into the PVA/PANI blend substantially improves *σ*_ac_. For instance, the pristine polymer blend shows an AC conductivity (*σ*_ac_) of 5.39 × 10^−8^ S cm^−1^ at high frequency, which rises to 3.32 × 10^−6^ S cm^−1^ at 4.5 wt% Fe_2_O_3_. This remarkable enhancement is attributed to the formation of additional charge transport pathways and a higher density of mobile carriers generated by the dispersed nanofiller. The Fe_2_O_3_ nanorods promote efficient charge movement by increasing the number of conduction channels and enabling long-range ion hopping. The XRD results further suggest that increased amorphous content provides localized states that facilitate ionic migration, while the recorded reduction in energy gap (UV/Vis.) implies a higher density of energy states, contributing to easier charge transfer.^50^ Overall, the results confirm that Fe_2_O_3_ incorporation effectively tunes the AC conductivity of PVA/PANI nanocomposites. A similar trend has been widely reported in various nanofiller-reinforced polymer systems, where a higher nanofiller loading results in enhanced electrical conductivity.^51^ The reported conductivity values correspond to the AC conductivity (*σ*_ac_) evaluated at a high-frequency region (*e.g.*, 10^6^ Hz), where the conductivity reaches its maximum and becomes less affected by electrode polarization effects. In addition, the DC conductivity (*σ*_dc_) was extracted from the frequency-independent plateau observed in the intermediate frequency region, as shown in Fig. 8 and summarized in Table 2.

#### 3.6.2 Dielectric parameters

Dielectric behavior plays a crucial role in determining the suitability of polymer-based materials for electrical insulation, charge storage, and next-generation flexible electronics. The complex dielectric permittivity of any dielectric medium is expressed as:8
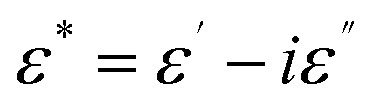
Here *ε*′ (dimensionless) corresponds to the energy stored inside the materials, and *ε*″(dimensionless) denotes the energy dissipated during each AC field cycle. These parameters can be determined through the following equations:^52^9
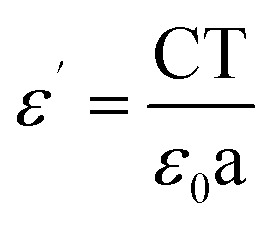
10
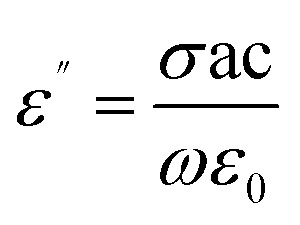


**Fig. 9:**
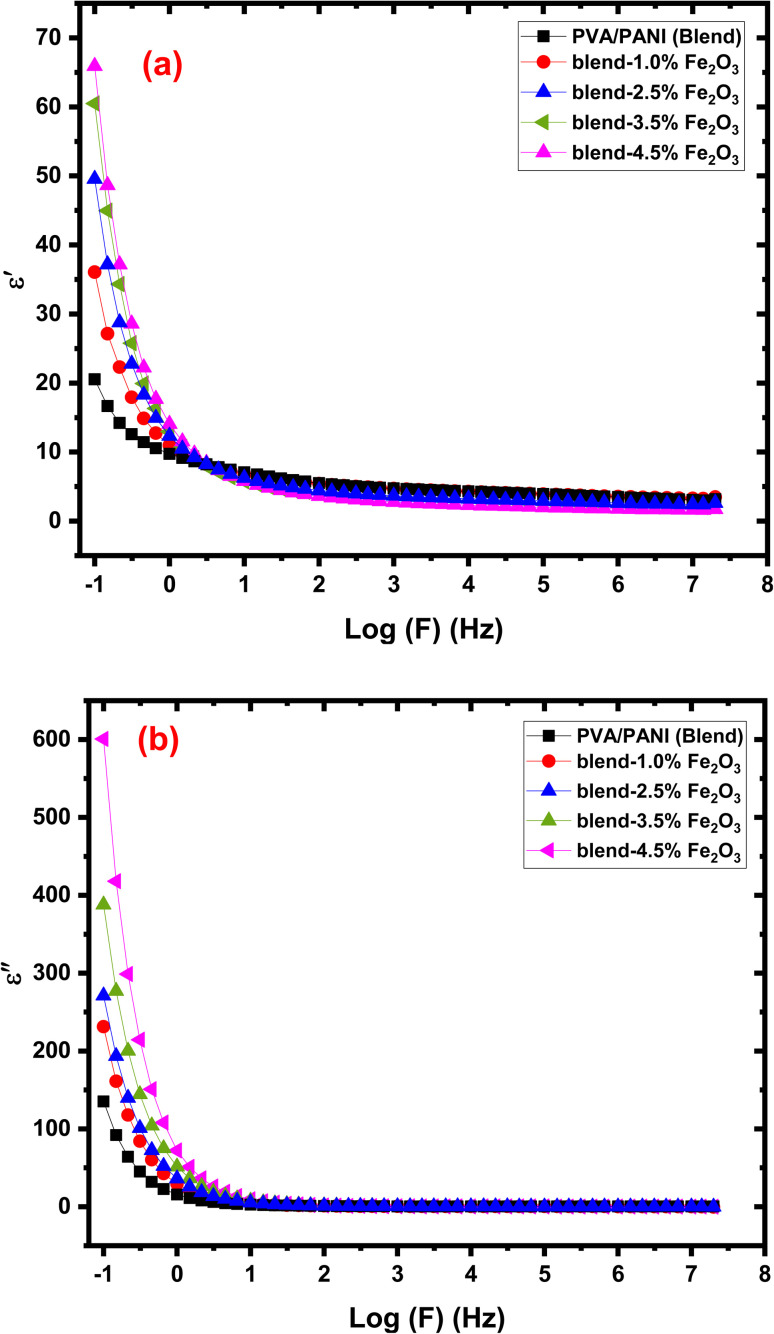
The plot of (a) *ε*′ and (b) *ε*″ with log (*f*) for the PVA/PANI-Fe_2_O_3_ nanocomposite samples.

Fig. 9(a and b) depicts the variation of the (*ε*′) and (*ε*″) permittivity parts with log (*f*) for PVA/PANI-Fe_2_O_3_ nanocomposites containing different filler contents. The values of (*ε*′) is strongly influenced by frequency due to Maxwell/Wagner interfacial polarizations. At lower frequencies, all samples exhibit high *ε*′ values as charges accumulate at the electrode–polymer interface, enabling dipoles to align effectively with the applied fields. When the frequency increases, dipole relaxation becomes delayed, reducing their ability to orient with the oscillating field, resulting in a gradual decline in *ε*′.^53,54^ At sufficiently high frequencies, only electronic and atomic polarization mechanisms remain active, while interfacial and orientational contributions gradually diminish. The observed enhancement in *ε*′ upon Fe_2_O_3_ incorporation is attributed to increased interfacial polarization sites and improved charge storage capability as a result of the creation of extra interfaces between the polymer and nanofiller.

The dielectric loss (*ε*″), shown in Fig. 9(b), also exhibits a strong frequency dependence. At low frequencies, *ε*″ values are high owing to the large segmental mobility of polymer chains and dominant dipolar polarization. As frequency rises, polymer segments can no longer follow the rapidly oscillating field, leading to a decrease in dielectric loss.^55^ An increase in *ε*″ with higher Fe_2_O_3_ concentration suggests stronger interfacial polarization and enhanced dipolar relaxation within the nanocomposite matrix. This effect is advantageous for energy-storage applications, such as capacitors, where high permittivity and charge retention are required.^56^ Conversely, the decreasing dielectric parameters at high frequencies indicate reduced energy dissipation, rendering these materials suitable for use as dielectric layers in micro- and nano-electronic systems.^57^

#### 3.6.3 Complex electric modulus

**Fig. 10:**
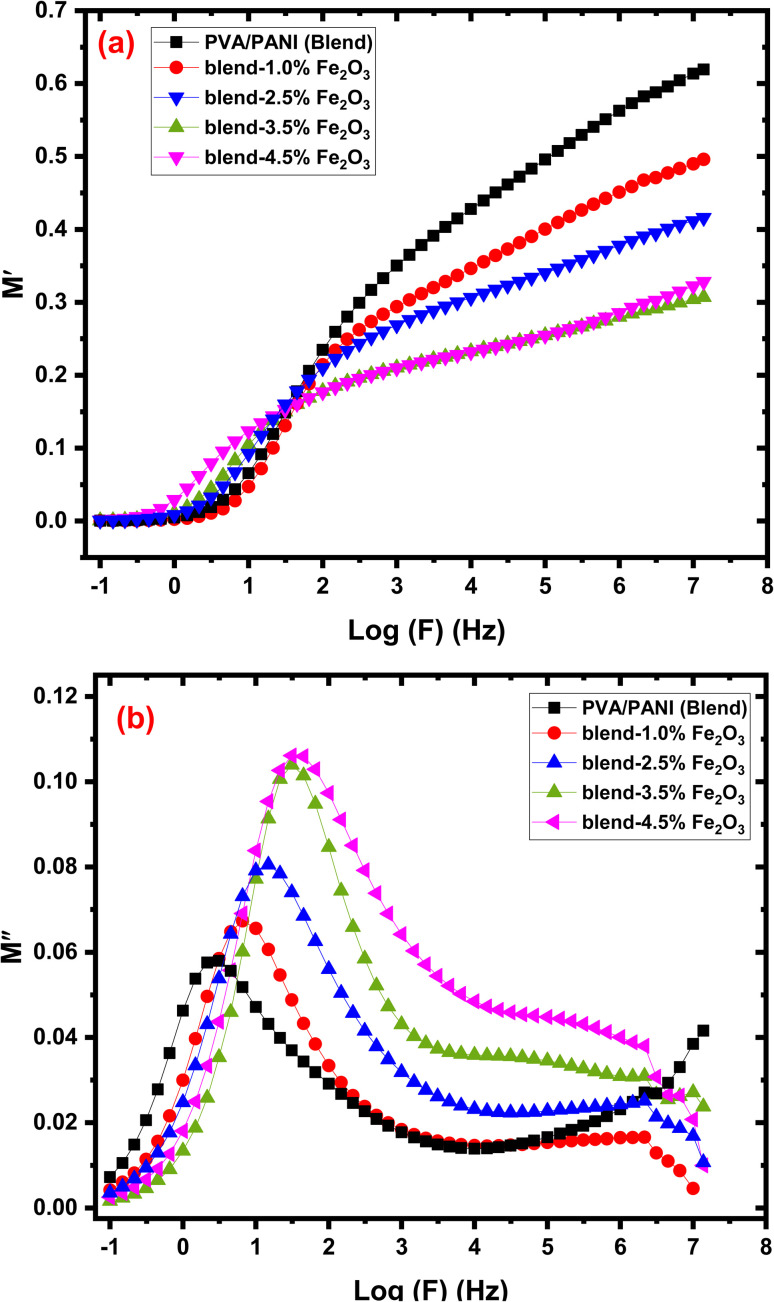
The plot of (a) *M*′ and (b) *M*″ with log (*f*) for the PVA/PANI-Fe_2_O_3_ nanocomposites samples.

The complex electric modulus (M*) is closely related to the dielectric permittivity (ε*) and is expressed as follows:^58,59^11
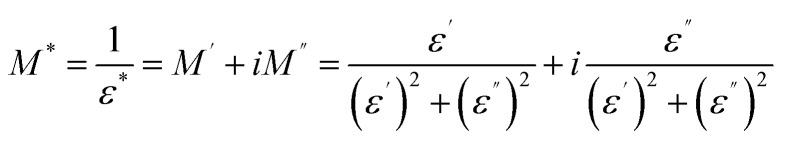
where *M*′ and *M*″ represent the real and imaginary components of the modulus, respectively. The modulus formalism is particularly valuable for analyzing relaxation dynamics and understanding ion-transport mechanisms in polymer nanocomposites. Fig. 10(a) and (b) display the variation of (*M*′) and (*M*″) with frequency for samples containing different Fe_2_O_3_ loadings. At low frequencies, both *M*′ and *M*″ approach zero, indicating negligible electrode polarization and limited charge-carrier behaviour within this frequency region. This behaviour suggests that charge transport is highly restricted and that the restoring force acting on the charge carriers is insufficient to generate polarization, implying that electrode effects do not dominate the relaxation process. As frequency increases, distinct and broadened peaks appear in the *M*′ and *M*″ spectra, demonstrating the occurrence of relaxation phenomena inside the nanocomposite system. These peaks correspond to the relaxation frequency (*f*_max_), which is inversely related to the relaxation time *τ* of mobile ions *via* the relation *τ* = 1/(2π*f*_max_). The existence of well-defined peaks confirms that ionic hopping is the primary conduction mechanism in these materials.^60,61^ The shift of the *M*″ peaks to higher frequencies with increasing Fe_2_O_3_ content indicates shorter relaxation times and enhanced ion mobility within the nanocomposite. The noticeably small *M*″ values at low frequencies additionally suggest minimal ion displacement and reduced polarization at the electrodes, consistent with restricted charge dynamics.^62^ Notably, the *M*″ peak intensity becomes higher upon incorporating Fe_2_O_3_ nanorods compared to the pristine PVA/PANI polymer blend, reflecting an altered relaxation time and enhanced ion mobility induced by the nanofiller. The broadening of the peaks suggests a distribution of relaxation times, likely due to heterogeneity introduced by the nanorods and local structural disorder. Overall, the electric modulus analysis confirms that α-Fe_2_O_3_ nanorods promote faster ion hopping and facilitate charge transport, consistent with the observed enhancement in AC conductivity.

#### 3.6.4 Impedance analysis

The electrical response of the prepared nanocomposites was examined using impedance spectroscopy, where both the real (*Z*′) and imaginary (*Z*″) components of impedance were determined according to the relations:^63^12
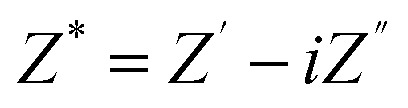
13
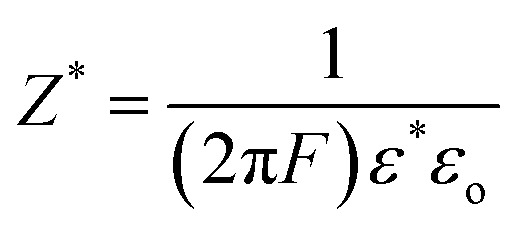


**Fig. 11:**
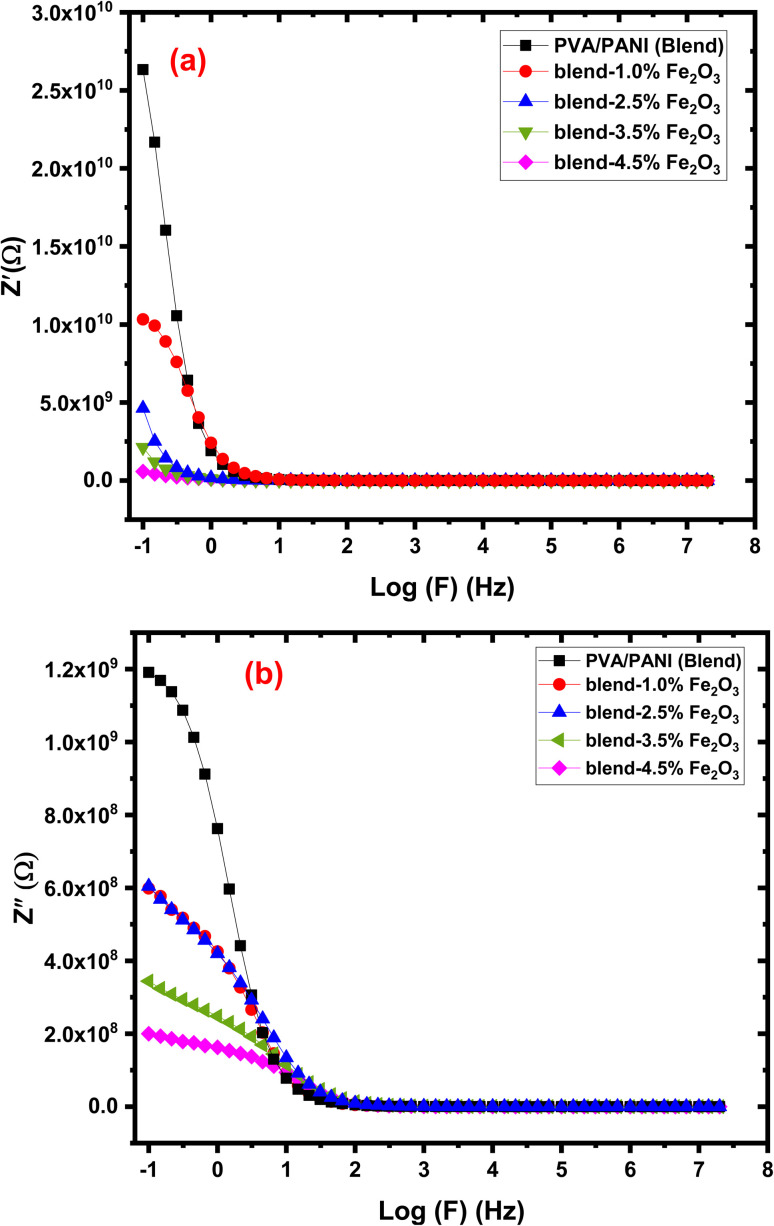
The plot of (a) *Z*′ and (b) *Z*″ with log (*f*) for the PVA/PANI-Fe_2_O_3_ nanocomposites samples.

The frequency dependent variations of (*Z′*) and (*Z*″) for PVA/PANI-Fe_2_O_3_ films are shown in Fig. 11(a and b). The impedance spectra provide essential insight into the charge-transport characteristics of the nanocomposites. Both components of impedance decrease with increasing frequency and ultimately merge at high frequencies, indicating diminished electrode polarization. In this high-frequency region, all Fe_2_O_3_-containing films exhibit lower *Z*′ values relative to the neat polymer blend, confirming improved charge transport and reduced resistance.^7^ A notable observation is the disappearance of the relaxation peak seen in pristine PVA/PANI. This peak is usually associated with space-charge accumulation; its suppression following nanofiller incorporation implies more effective charge transfer and enhanced capacitive behavior. Increasing Fe_2_O_3_ content further reduces *Z*″, suggesting the formation of additional conduction pathways throughout the composite structure. The sample containing 4.5 wt% Fe_2_O_3_ shows the lowest *Z*′, verifying that Fe_2_O_3_ nanorods significantly enhance carrier mobility within the matrix.^55^ These results reveal that PVA/PANI-Fe_2_O_3_ nanocomposites possess superior electrical performance compared to the pure polymer system. Their high ionic conductivity, reduced impedance, and effective charge dynamics make them excellent candidates for energy-storage applications, particularly in high-frequency dielectric capacitor technologies.^64^

#### 3.6.5 Nyquist analysis

**Fig. 12:**
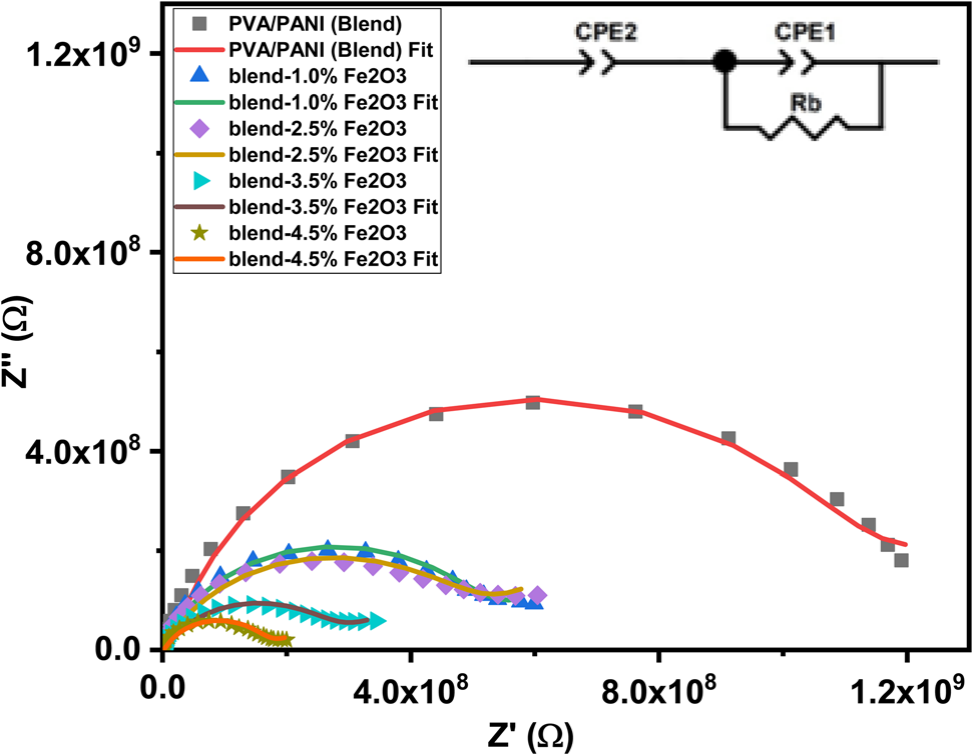
The relation between (*Z*′) and (*Z*″) for the PVA/PANI-Fe_2_O_3_ nanocomposites samples.

Nyquist plots of the PVA/PANI-Fe_2_O_3_ nanocomposites exhibit a characteristic semicircular arc in the higher frequencies range and subsequently showing a linear tail at low frequency, reflecting two distinct electrochemical processes, as shown in Fig. 11. The impedance spectra were interpreted using an equivalent electrical circuit model consisting of the bulk resistance (*R*_b_) in parallel with two constant phase elements (CPE1 and CPE2). The value of *R*_b_, obtained from the intercept of the semicircle with the *Z*′ axis, represents the intrinsic resistance of the material.^65,66^ The high-frequency semicircle corresponds to the bulk response of the system, where *R*_b_ and CPE1 dominate the relaxation process. CPE1 accounts for interfacial effects at the electrode–electrolyte boundary and participates directly in charge-transfer mechanisms. In contrast, the low-frequency inclined line is associated with diffusion-controlled transport or a more complex relaxation that cannot be described by *R*_b_ and CPE alone. A progressive decrease in the diameter of the semicircle with increasing Fe_2_O_3_ content confirms a reduction in bulk resistance and enhanced electrical conductivity.^67^ This improvement arises from the formation of new ion-transport pathways facilitated by the nanofiller, which reduces the activation energy required for ionic motion.^68^ The finding aligns well with FTIR and XRD analyses, which verify strong interactions between the filler and polymer chains. The addition of Fe_2_O_3_ significantly boosts ionic conductivity by promoting more continuous conduction channels along the polymer network, enabling easier ion migration.^69^ The parameters (*Q*_1_), (*n*_1_), (*Q*_2_), and (*n*_2_) corresponding to (CPE1) and (CPE2) were extracted through circuit fitting (Table 3) to provide quantitative insight into relaxation dynamics within the system. A marked decrease in *R*_b_ [from 1.00 × 10^9^ Ω to 1.51 × 10^8^ Ω] was observed at high filler loading, indicating the presence of more efficient conduction pathways and improved ion mobility under alternating electric fields, a behavior consistent with semiconductor conduction mechanisms.^70^ Meanwhile, the shrinking semicircle at high frequency reflects reduced impedances at interfaces and higher carrier mobility Fig. 12. Collectively, these features demonstrate that Fe_2_O_3_-enriched PVA/PANI nanocomposites possess favorable conductivity, strong capacitive characteristics, and high charge-storage efficiency.^71^

**Table 3:** Optimized circuit-model fitting parameters for the PVA/PANI-Fe_2_O nanocomposites

Composites	Fitting parameters				
	*R*_b_ (Ω)	*Q*_1_ (F)	*n*_1_	*Q*_2_ (F)	*n*_2_
PVA/PANI (blend)	1.00 × 10^9^	1.61 × 10^−10^	0.93	4.72 × 10^−9^	0.37
Blend-1.0% Fe_2_O_3_	4.99 × 10^8^	1.69 × 10^−10^	0.83	1.08 × 10^−8^	0.46
Blend-2.5% Fe_2_O_3_	4.72 × 10^8^	1.72 × 10^−10^	0.78	8.14 × 10^−9^	0.45
Blend-3.5% Fe_2_O_3_	2.51 × 10^8^	2.44 × 10^−10^	0.74	1.21 × 10^−8^	0.35
Blend-4.5% Fe_2_O_3_	1.51 × 10^8^	2.01 × 10^−10^	0.79	2.21 × 10^−8^	0.28

## 4 Conclusion

This study presents a comprehensive evaluation of PVA/PANI-Fe_2_O_3_ nanocomposites, demonstrating the strong influence of Fe_2_O_3_ nanorod loading on their structural, optical, and electrical properties. XRD and FTIR confirmed the successful incorporation of Fe_2_O_3_ into the PVA/PANI matrix, leading to reduced crystallinity, increased disorder, and strong filler–polymer interactions. Optical assessment revealed a red shift in absorption spectra along with significant band gap narrowing (*E*_gd_: 3.91 → 2.59 eV; *E*_gi_: 2.45 → 1.09 eV), while Urbach energy increased, confirming enhanced defect-state density and improved electronic transition capability. The nanocomposites also exhibited substantial enhancement in AC electrical conductivity from 5.39 × 10^−8^ to 3.32 × 10^−6^ S cm^−1^, attributed to improved charge carrier mobility and interfacial polarization. Overall, increasing Fe_2_O_3_ content effectively tunes the optical band structure, increases electrical conductivity, and improves dielectric response, positioning these nanocomposites as strong candidates for next-generation optoelectronic, photonic, and energy-related devices. This work demonstrates that the incorporation of α-Fe_2_O_3_ nanorods, rather than conventional nanoparticles, plays a key role in enhancing charge transport and tailoring the optical band gap. The combined improvement in optical and electrical properties highlights the potential of these nanocomposites for optoelectronic applications.

## Author contributions

H. M. Ragab: methodology, formal analysis, investigation, writing – review and editing. N.S. Diab: investigation, writing – review & editing Rosilah Ab Aziz: methodology, formal analysis, investigation. Shimaa Mohammed Aboelnaga: investigation, writing – review & editing. Tahani M. Alresheedi: investigation, writing – review & editing. M.O. Farea: conceptualization, methodology, writing – review & editing. A Al Ojeery: investigation, writing – review & editing.

## Conflicts of interest

The authors declare no conflicts of interest.

## Data Availability

The datasets generated and/or analyzed during the current study are available from the corresponding author on reasonable request.
